# Genomic Landscape Features of Minimally Invasive Adenocarcinoma and Invasive Lung Adenocarcinoma

**DOI:** 10.1055/s-0044-1791198

**Published:** 2024-09-19

**Authors:** Wei Zhang, Hui Xu, Ning Tang, Shuang Han, Hongyan Shu

**Affiliations:** 1 Department of Thoracic Surgery, Zibo Municipal Hospital, Zibo, Shandong, China; 2 Department of Anesthesiology, Zibo Municipal Hospital, Zibo, Shandong, China; 3 Department of Stomatology, Zibo Municipal Hospital, Zibo, Shandong, China; 4 Department of Endocrinology, Zibo Municipal Hospital, Zibo, Shandong, China

**Keywords:** lung cancer, minimally invasive adenocarcinoma, invasive adenocarcinoma, mutations

## Abstract

**Background** The widespread implementation of computed tomography has significantly increased the detection of small pulmonary nodules, including atypical adenomatous hyperplasia, minimally invasive adenocarcinoma (MIA), and invasive adenocarcinoma (IAC). Few studies have focused on the genomic differences between MIA and IAC.

**Methods** We retrospectively analyzed patients with lung adenocarcinoma (LUAD) who underwent surgery from January 2020 to December 2023. Patients were categorized into MIA and IAC groups. The mutation status of common driver genes was assessed using next-generation sequencing.

**Results** A total of 422 LUAD patients were included in the study, comprising 119 MIA cases and 303 IAC cases. MIA patients were younger and predominantly female compared with IAC patients. EGFR mutations were detected in 251 patients (59.5%), with the frequency of EGFR mutations increasing from 37.0% in MIA to 68.3% in IAC ( *p* < 0.001). TP53 mutations were found in 108 patients (25.6%), with 7 patients (5.9%) in MIA and 101 patients (33.3%) in IAC ( *p* < 0.001). ERBB2 mutations were identified in 23 MIA patients (19.3%) and 20 IAC patients (6.6%) ( *p* < 0.001). Additionally, CDKN2A mutations were detected in 23 IAC patients (7.6%), while no mutations in this gene were found in the MIA group. Moreover, ALK and RET gene fusions were identified in 11 patients, respectively.

**Conclusion** ERBB2 mutations and RET fusions are early genomic events in LUAD, while TP53 and CDKN2A mutations and ALK fusions occur later. Genomic intratumor heterogeneity likely arises early, before invasive characteristics develop.

## Introduction

Lung cancer is one of the most prevalent cancers globally and remains the leading cause of cancer-related mortality among all human malignancies. 1 Both developing and developed countries have observed significant increases in lung cancer morbidity and mortality rates, with particularly notable rises in China. 2 The widespread use of low-dose spiral computed tomography in health checkups has led to the detection of an increasing number of lung nodules. 3 Among these malignant lung nodules, lung adenocarcinoma (LUAD) is the predominant histopathological type, ranging from adenocarcinoma in situ (AIS) to minimally invasive adenocarcinoma (MIA) and invasive adenocarcinoma (IAC), illustrating a gradual progression trend. 4 5 The dynamic evolution from AIS to MIA and IAC is a current research focus; however, the results thus far have been unsatisfactory.

The diagnosis of early-stage LUAD has increased, with Stage I LUAD, including MIA, being the most prevalent type in China. 6 However, research on the characteristics of driver gene mutations and their significance in lung MIA and IAC remains limited. Histological progression is associated with progressive DNA alterations, including an increased mutational burden characterized by the accumulation of single-nucleotide variants (SNVs), somatic copy number alterations, and mutations in canonical oncogenes such as *EGFR* , *KRAS* , and *TP53* . 7 8 9

Previous studies have been limited by small sample sizes, restricting the statistical significance of their findings. This limitation has motivated us to investigate the molecular basis of tumor behavior in early-stage cancer using a larger population. Our goal is to gain insights into the molecular characteristics and underlying determinants of clinical behavior in early LUAD. In this study, we describe the spectrum of driver gene mutations in MIA and IAC specimens from LUAD patients.

## Materials and Methods

### Clinical Specimens

Formalin-fixed paraffin-embedded (FFPE) specimens from LUAD patients who underwent next-generation sequencing (NGS) between 2021 and 2023 at Yinfeng Gene Technology Co., Ltd. were included in this study. The diagnosis of the specimens was confirmed by hematoxylin and eosin staining performed by an independent pathologist. For further analysis, the specimens were required to have a tumor cell percentage of more than 20% and a size of ≥1 mm.

### NGS Sequencing

DNA extracted from the FFPE tumor specimens was sequenced on an NGS platform using a comprehensive 500-cancer gene panel with the Illumina cBot Cluster Generation System (Illumina, Inc.). This panel includes key driver genes and mutations relevant to LUAD, providing sufficient molecular information for analyzing the mutational characteristics of MIA and IAC. The DNA libraries were sequenced on an Illumina HiSeq 2000 system (Illumina, Inc.). Genetic alterations were identified, microsatellite instability (MSI) status was assessed, and clinical information, including age, gender, and tumor histology, was collected. Germline variants were identified by comparing the patient's tumor DNA to matching blood controls. The detected genetic alterations included SNVs, insertions/deletions (indels), copy number variations, and gene rearrangements.

### Statistical Analysis

SPSS software was used for statistical analysis. Fisher's exact test and one-way analysis of variance were employed to analyze associations between clinical data and genetic characteristics. The *p* -values were two-sided, with *p* < 0.05 considered statistically significant unless otherwise specified.

## Results

### Clinical Characteristics of the Patients

**Fig. 1:**
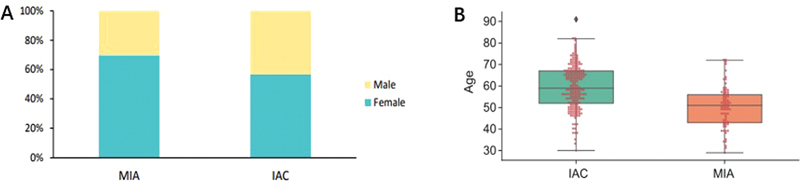
Age and gender differences in MIA and IAC patients. ( **A** ) Gender. ( **B** ) Age. IAC, invasive adenocarcinoma; MIA, minimally invasive adenocarcinoma.

This study included a total of 422 patients, of whom 119 were diagnosed with MIA and 303 with IAC. Clinical information, including age, sex, MSI status, and tumor proportion score expression, was collected. Among the MIA patients, 83 (69.7%) were women and 36 (30.3%) were men, with a median age of 51 years. In the IAC group, 172 (56.8%) were women and 131 (43.2%) were men, with a median age of 59 years. Significantly more female and younger patients were identified in the MIA group compared with the IAC group ( *p* < 0.001) ( Fig. 1A, B ).

Among the 119 patients with MIA, 3 (2.5%) had high MSI status, while among the 303 IAC patients, 13 (4.3%) had high MSI status. The cutoff for MSI was 29 (13.5%) based on the evaluation of 55 microsatellite markers. The programmed death-ligand 1 (PD-L1) positive rate in LUAD from MIA to IAC stage is shown in Table 1 . Two (1.7%) of the MIA cases showed strongly positive PD-L1 expression (PD-L1 ≥ 50%), and 21 (17.6%) showed positive PD-L1 expression (1% ≤ PD-L1 < 50%). In IAC LUAD, the percentages of PD-L1-positive samples were 8 (2.6%, PD-L1 ≥ 50%) and 69 (22.8%, 1% ≤ PD-L1 < 50%). Interestingly, PD-L1 expression increased with the invasiveness of LUAD.

**Table 1:** Clinicopathological information of MIA and IAC patients

Characteristics	MIA ( *n* = 119)	IAC ( *n* = 303)
Sex, *n* (%)		
Male	36 (30.3%)	131 (43.2%)
Female	83 (69.7%)	172 (56.8%)
Age, median (range)	51 (43–56)	59 (52–67)
MSI status, *n* (%)		
High MSI	3 (2.5%)	13 (4.3%)
MSS	116 (97.5%)	290 (95.7%)
PD-L1		
PD-L1 < 1%	37 (31.1%)	103 (34.0%)
1% ≤ PD-L1 < 50%	21 (17.6%)	69 (22.8%)
PD-L1 ≥ 50%	2 (1.7%)	8 (2.6%)
N/A	59 (49.6%)	123 (40.6%)

Abbreviations: IAC, invasive adenocarcinoma; MIA, minimally invasive adenocarcinoma; MSI, microsatellite instability; MSS, microsatellite stability; N/A, not available; PD-L1, programmed death-ligand 1.

### Mutation Landscape of MIA and IAC Patients

**Fig. 2:**
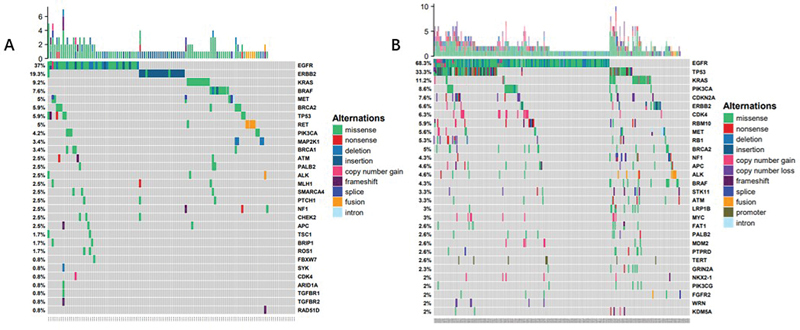
The high-frequency mutation genes in ( **A** ) MIA and ( **B** ) IAC patients. IAC, invasive adenocarcinoma; MIA, minimally invasive adenocarcinoma.

**Fig. 3:**
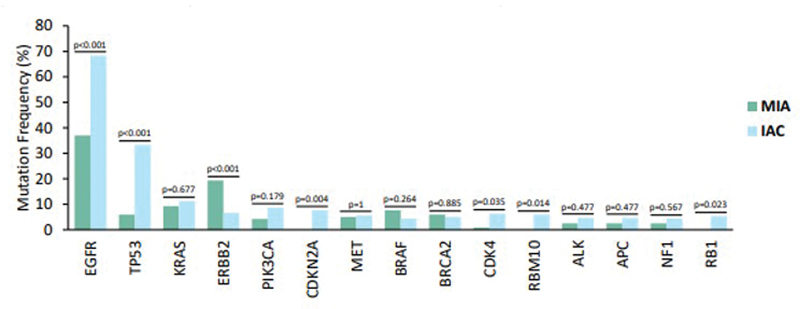
Differences of the high-frequency mutation genes between MIA and IAC patients. IAC, invasive adenocarcinoma; MIA, minimally invasive adenocarcinoma.

In patients with MIA, high-frequency mutations included EGFR (37%), ERBB2 (19.3%), KRAS (9.2%), BRAF (7.6%), MET (5%), TP53 (5.9%), BRCA2 (5.9%), RET (5%), and PIK3CA (4.2%) ( Fig. 2A ). In patients with IAC, the most frequent mutations were EGFR (68.3%), TP53 (33.3%), KRAS (11.2%), PIK3CA (8.6%), CDKN2A (7.6%), ERBB2 (6.6%), and CDK4 (6.3%) ( Fig. 2B ). Compared with IAC patients, significantly more mutations were observed in ERBB2 ( *p* < 0.001) and BRAF ( *p* = 0.264) in the MIA group, while fewer mutations were observed in EGFR ( *p* < 0.001). The higher frequency of TP53 mutations in the IAC group aligns with their occurrence in later stages of disease progression. CDKN2A mutations were significantly more frequent in IAC patients than in MIA patients ( *p* = 0.004). No significant differences in KRAS mutations were noted between the two groups. These findings suggest that the frequency of TP53, EGFR, and CDKN2A mutations may increase with the infiltration and progression of LUAD ( Fig. 3 ).

**Fig. 4:**
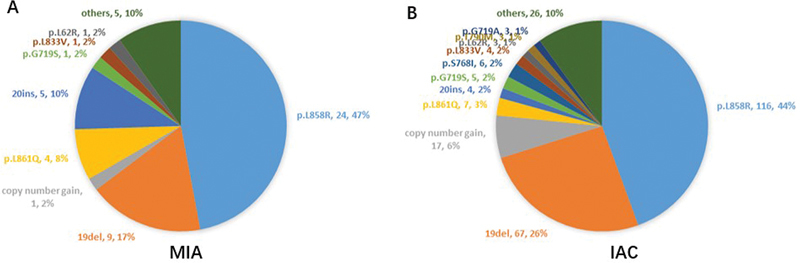
Mutation spectrum of the epidermal growth factor receptor gene in ( **A** ) MIA and ( **B** ) IAC patients. IAC, invasive adenocarcinoma; MIA, minimally invasive adenocarcinoma.

EGFR mutation status was analyzed, revealing mutations in 251 patients (59.5%). Notably, the frequency of EGFR mutations increased from 37.0% in MIA to 68.3% in IAC ( *p* < 0.001) ( Fig. 3 ). The most common mutation types were exon 19 deletions and the L858R mutation in exon 21. Among MIA patients, exon 19 deletions were observed in 9 (17%) patients, and the L858R mutation in 24 (47%) patients. In IAC patients, these mutations were found in 67 (26%) and 116 (44%) patients, respectively. The proportions of L858R and exon 19 deletions among all EGFR mutations did not significantly differ between the subtypes. However, the frequency of the EGFR 20ins mutation was significantly higher in IAC patients compared with MIA patients (10 vs. 2%) ( Fig. 4 ).

### ALK and RET Fusions of MIA and IAC Patients

ALK and RET gene fusions are significant driver genes in lung adenocarcinoma, and targeted therapies have been approved for these alterations in lung cancer. Among the 119 MIA patients, only one patient (0.8%) of ALK fusion was identified. However, in the IAC cohort, the frequency of ALK fusion increased to 3.3% (10/303), suggesting that ALK fusions are more common in advanced-stage lung cancer. Notably, no patients were found to have both EGFR mutations and ALK fusions. Furthermore, RET gene fusions were identified in seven patients (5.9%) in the MIA cohort, while in the IAC cohort, RET fusions were identified in only four patients (1.3%) ( Table 2 ).

**Table 2:** Different forms of ALK and RET fusions in MIA and IAC patients

	Fusion site	MIA number	IAC number
ALK fusion	EML4-ALK (exon 13: exon 20)		4
	EML4-ALK (exon 20: exon 20)		1
	EML4-ALK (exon 6: exon 20)	1	1
	EML4-ALK (exon 7: exon 20)		1
	KLC1-ALK (exon 9: exon 20)		1
	ALK-CDC42EP3 (exon 19: promoter)		1
	ALK-LINC00301 (exon 20: exon 6)		1
RET fusion	RET-PRKG1 (exon 10: exon 11)	1	
	KIF5B-RET (exon 23: exon 12)	1	
	KIF5B-RET (exon 15: exon 12)	1	1
	KIF5B-RET (exon 15: exon 11)		1
	CCDC6-RET (exon 1: exon 12)	1	
	APAF1-RET (exon 5: exon 12)	1	
	CPEB2-RET (intergenic: exon 12)	1	
	CXCL12-RET (intergenic: exon 8)		1
	RET-RPIA (exon 7: intergenic)		1
	RET-TMPO (exon 11: intergenic)	1	

### Germline Mutations of MIA and IAC Patients

Emerging data highlight the existence of additional, previously undescribed pathogenic germline variants (PGVs) as causative etiologies in non-small cell lung cancer (NSCLC). PGVs in cancer susceptibility genes may predispose individuals to tumorigenesis. Expanding evidence demonstrates that patients harboring certain PGVs have an increased risk of developing lung cancer. 10 In our study cohort, 9 out of 303 (3.0%) IAC patients were found to have germline variants annotated as pathogenic or likely pathogenic in ClinVar. These nine IAC patients harbored heterozygous mutations in genes including *WRN* , *PALB2* , *MRE11A* , *BRCA1* , *RAD51D* , *BRCA2* , *CHEK2* , and *ATM* ( Table 3 ). Notably, these germline mutations were not detected in any of the 119 MIA patients.

**Table 3:** Germline mutations in nine IAC patients

Patient no.	Sex	Age	Cancer type	Gene	C.	P.	Mutation type	Clinical significance	Evidence source
1	Female	67	IAC	*WRN*	c.182delA	p.D61Vfs*11	Frameshift	Pathogenic	ClinVar
2	Female	49	IAC	*PALB2*	c.3228delT	p.C1078Vfs*17	Frameshift	Pathogenic	ClinVar
3	Female	71	IAC	*MRE11A*	c.25delG	p.D9Mfs*8	Frameshift	Pathogenic	ClinVar
4	Male	68	IAC	*BRCA1*	c.3294delT	p.P1099Lfs*10	Frameshift	Pathogenic likely	ClinVar
5	Male	73	IAC	*RAD51D*	c.270_271dupTA	p.K91Ifs*13	Frameshift	Pathogenic likely	ClinVar
6	Male	66	IAC	*RAD51D*	c.898C > T	p.R300*	Nonsense	Pathogenic likely	ClinVar
7	Male	NA	IAC	*BRCA2*	c.2059_2063delGATTA	p.D687*fs*1	Frameshift	Pathogenic likely	ClinVar
8	Female	NA	IAC	*CHEK2*	c.847-14_847-2delinsGG		Splice	Pathogenic likely	ClinVar
9	Female	57	IAC	*ATM*	Deletion		Copy number loss	Pathogenic likely	ClinVar

Abbreviation: IAC, invasive adenocarcinoma.

## Discussion

Large-scale sequencing studies have elucidated the complex genomic landscape of NSCLC. 11 In clinical practice, routine detection of driver gene mutations is not recommended for MIA patients due to their infrequent recurrence and metastasis. However, emerging evidence from sporadic studies suggests that driver gene mutations may play a significant role in the progression of LUAD from precancerous lesions to IAC. 12 13 MIA is a newly defined subtype of LUAD with distinctive clinicopathological features. 14 It is a common type of stage I lung cancer, and its prognosis remains controversial. Previous studies have shown that lung MIA occurs more frequently in females, never smokers, and patients aged 51 to 60 years. 15 This is consistent with our findings, where the majority of MIA patients were relatively young females.

The detection of EGFR mutations is becoming a prerequisite for tailored treatment in late-stage NSCLC. With the increasing diagnosis of early-stage LUAD, stage I LUAD, including MIA, has become the most common type in China. 7 Previous studies have indicated that EGFR mutations are observed more frequently (40–60%) in LUADs among Asian patients. 16 17 Additionally, the current study revealed that 19Del and L858R are the two predominant mutation subtypes, consistent with previous findings in patients with IAC. 18 The role of driver gene status in MIA or IAC remains a subject of debate. Zhu et al suggested that the increased frequency of EGFR mutations might drive the progression from AIS to MIA (33.3 vs. 50.8%), while the frequency of EGFR mutations remained relatively stable from MIA to IAC (50.8 vs. 50.2%). 9 Consistent with our findings, Kobayashi et al reported that EGFR gene detection was performed on 104 ground-glass nodules specimens from 96 patients, showing that EGFR mutation-positive tumors were associated with growth as they progressed from AIS to MIA to IAC. 19 In our study, the increased frequency of EGFR mutations appears to have driven the progression from MIA to IAC (37.0 vs. 68.3%, *p* < 0.001). These findings suggest that EGFR mutations may represent early molecular events in the carcinogenesis of LUAD. The accumulation of driver gene mutations likely initiates the transition from MIA to IAC in LUAD. Our study has identified several key genomic alterations that differentiate MIA from IAC, and these findings have important clinical correlations. For instance, mutations in genes such as *EGFR* and *ALK* , which are more prevalent in IAC, are known to be associated with specific targeted therapies. Patients with these mutations may benefit from EGFR tyrosine kinase inhibitors or ALK inhibitors, which have been shown to improve outcomes in this patient population.

In addition, our work reveals that the frequency of TP53 mutations is significantly higher in IAC than in MIA, suggesting that TP53 mutations are relatively later molecular events driving tumor progression. Previous studies in Barrett's esophagus have suggested that TP53 mutations occur early in esophageal adenocarcinoma precursors, followed by oncogenic amplifications. 20 TP53 mutations are also frequently found in lung carcinoma in situ, the precursor form of squamous cell carcinoma. 21 Earlier studies have shown a high frequency of oncogenic driver mutations but a low frequency of TP53 mutations in LUAD precursors. 22 Other studies have suggested a functional association between TP53 mutations and invasive potential in cancers. 23 Therefore, it is speculated that TP53 acts as a key mediator in the invasiveness of lung cancer. Furthermore, the presence of TP53 mutations, often associated with more aggressive tumor behavior, suggests a potential for poor prognosis and may guide the choice of more aggressive treatment strategies in affected patients. In contrast, the relatively lower mutation burden in MIA might correlate with a more indolent disease course, supporting the use of less aggressive treatment approaches.

In this study, we have identified several key mutations that differentiate MIA from IAC. The clinical significance of these mutations is substantial, as they could potentially guide treatment decisions and influence patient outcomes. For instance, mutations in genes such as *EGFR* , *ALK* , and *CDKN2A* , which are frequently observed in IAC, are known to have direct implications for targeted therapies. The presence of these mutations may inform the selection of appropriate molecularly targeted agents, such as tyrosine kinase inhibitors, and help predict responses to therapy. Moreover, the identification of certain mutations may also provide insights into resistance mechanisms, offering opportunities to adjust treatment strategies preemptively. In the context of MIA, where fewer driver mutations were observed, the implications for treatment may be different, potentially allowing for less aggressive therapeutic approaches. Overall, our findings underscore the importance of integrating genomic profiling into the clinical management of LUAD, enabling more personalized and effective treatment plans.

While our study provides a comprehensive analysis of the genomic landscape in MIA and IAC, we recognize that functional studies are essential to confirm the biological roles of the specific mutations identified. Techniques such as CRISPR/Cas9 gene editing or RNA interference could be employed to validate the contribution of these mutations to tumor progression and metastasis. For instance, a recent study demonstrated the utility of these approaches in elucidating the functional impact of genetic alterations in cancer. 24 We suggest that future research should incorporate these functional studies to provide a more detailed understanding of how specific genomic alterations contribute to the clinical behavior of MIA and IAC. This would not only confirm the findings of our genomic analysis but also potentially identify novel therapeutic targets for more effective treatment strategies.

During the sequencing process, several challenges were encountered that required careful consideration and management. One of the primary challenges was ensuring the quality of the tumor samples, as degradation or contamination could significantly affect the accuracy of the sequencing results. To address this, we implemented stringent quality control measures, including the assessment of DNA integrity before sequencing. Another challenge was achieving sufficient sequencing depth to reliably detect low-frequency mutations, particularly in heterogeneous tumor samples. We employed high-coverage sequencing strategies and bioinformatics tools to enhance the detection sensitivity and ensure that rare variants were not overlooked. Additionally, the interpretation of complex genomic data posed a challenge, especially in distinguishing between driver mutations and passenger mutations. To overcome this, we utilized multiple databases and functional prediction tools to validate the clinical relevance of the identified mutations. Despite these challenges, the robustness of our methodology allowed us to generate reliable and meaningful insights into the genomic landscape of MIA and invasive LUAD.

In this study, we have identified key genomic features that differentiate MIA from IAC. However, several limitations should be acknowledged. First, the study's sample size, while sufficient to draw significant conclusions, may limit the generalizability of our findings to broader populations. Second, the study focused primarily on genomic data, without the inclusion of other omics data such as transcriptomics or proteomics, which could offer a more comprehensive understanding of the molecular mechanisms at play. Finally, the cross-sectional design of the study limits our ability to conclude the temporal progression from MIA to IAC. Future studies addressing these limitations will be critical to further elucidate the genomic and molecular characteristics of these LUAD subtypes.
